# Degradation of dibutyl phthalate by *Paenarthrobacter* sp. Shss isolated from Saravan landfill, Hyrcanian Forests, Iran

**DOI:** 10.1007/s10532-021-09966-7

**Published:** 2021-11-09

**Authors:** S. Shariati, C. Ebenau-Jehle, A. A. Pourbabaee, H. A. Alikhani, M. Rodriguez-Franco, M. Agne, M. Jacoby, R. Geiger, F. Shariati, M. Boll

**Affiliations:** 1 Faculty of Biology, Microbiology, University of Freiburg, Freiburg, Germany; 2 Department of Soil Science Engineering, University of Tehran, Tehran, Iran; 3 Faculty of Biology, Cell Biology, University of Freiburg, Freiburg, Germany; 4 Spemann Graduate School of Biology and Medicine (SGBM), University of Freiburg, Freiburg, Germany; 5 Department of Environmental Science, Islamic Azad University, Lahijan, Iran

**Keywords:** Phthalic acid ester, *Ortho*-phthalic acid, Microbial degradation, Bioremediation, Esterase

## Abstract

Phthalic acid esters are predominantly used as plasticizers and are industrially produced on the million ton scale per year. They exhibit endocrine-disrupting, carcinogenic, teratogenic, and mutagenic effects on wildlife and humans. For this reason, biodegradation, the major process of phthalic acid ester elimination from the environment, is of global importance. Here, we studied bacterial phthalic acid ester degradation at Saravan landfill in Hyrcanian Forests, Iran, an active disposal site with 800 tons of solid waste input per day. A di-*n*-butyl phthalate degrading enrichment culture was established from which *Paenarthrobacter* sp. strain Shss was isolated. This strain efficiently degraded 1 g L^–1^ di-*n*-butyl phthalate within 15 h with a doubling time of 5 h. In addition, dimethyl phthalate, diethyl phthalate, mono butyl phthalate, and phthalic acid where degraded to CO_2_, whereas diethyl hexyl phthalate did not serve as a substrate. During the biodegradation of di-*n*-butyl phthalate, mono-*n*-butyl phthalate was identified in culture supernatants by ultra-performance liquid chromatography coupled to electrospray ionization quadrupole time-of-flight mass spectrometry. In vitro assays identified two cellular esterase activities that converted di-*n*-butyl phthalate to mono-*n*-butyl phthalate, and the latter to phthalic acid, respectively. Our findings identified *Paenarthrobacter* sp. Shss amongst the most efficient phthalic acid esters degrading bacteria known, that possibly plays an important role in di-*n*-butyl phthalate elimination at a highly phthalic acid esters contaminated landfill.

## Introduction

Esters of *ortho*-phthalic acid (*o*-phthalic acid, 1,2-benzenedicarboxylic acid) and various alcohols are termed phthalic acid esters, often simply referred to as phthalates. Phthalic acid esters (PAEs) are industrially produced as plasticizers to enhance the flexibility of various plastic polymer compounds. They are found in a variety of products such as paints, polishers, lubricants, adhesives, pulp and paper industries, children’s toys, and cosmetic products. Introduced in the 1930s, the annual global production of PAE-derived plasticizers now reaches the million ton scale accounting for 65–70% of the total plasticizer products (Baloyi et al. 2021). PAEs are non-covalently bound to plastic polymers and can easily leach from polymer matrices into the environment during manufacturing, use and disposal (Liang et al. 2008; Net et al. 2015; Gao and Wen 2016; Boll et al. 2020).

PAEs belong to a group of endocrine disrupting chemicals with numerous adverse effects on wildlife (Oehlmann et al. 2009) and human health including reproductive disorders, allergic diseases, breast cancer, testosterone level reduction, obesity, and diabetes (recently reviewed in Kahn et al. 2020). A wealth of studies have identified PAEs as contaminants in soil, surface water, sediments, seawater, municipal wastewater and landfill leachate (Liang et al. 2008; Net et al. 2015; Gao and Wen 2016).

**Fig. 1:**
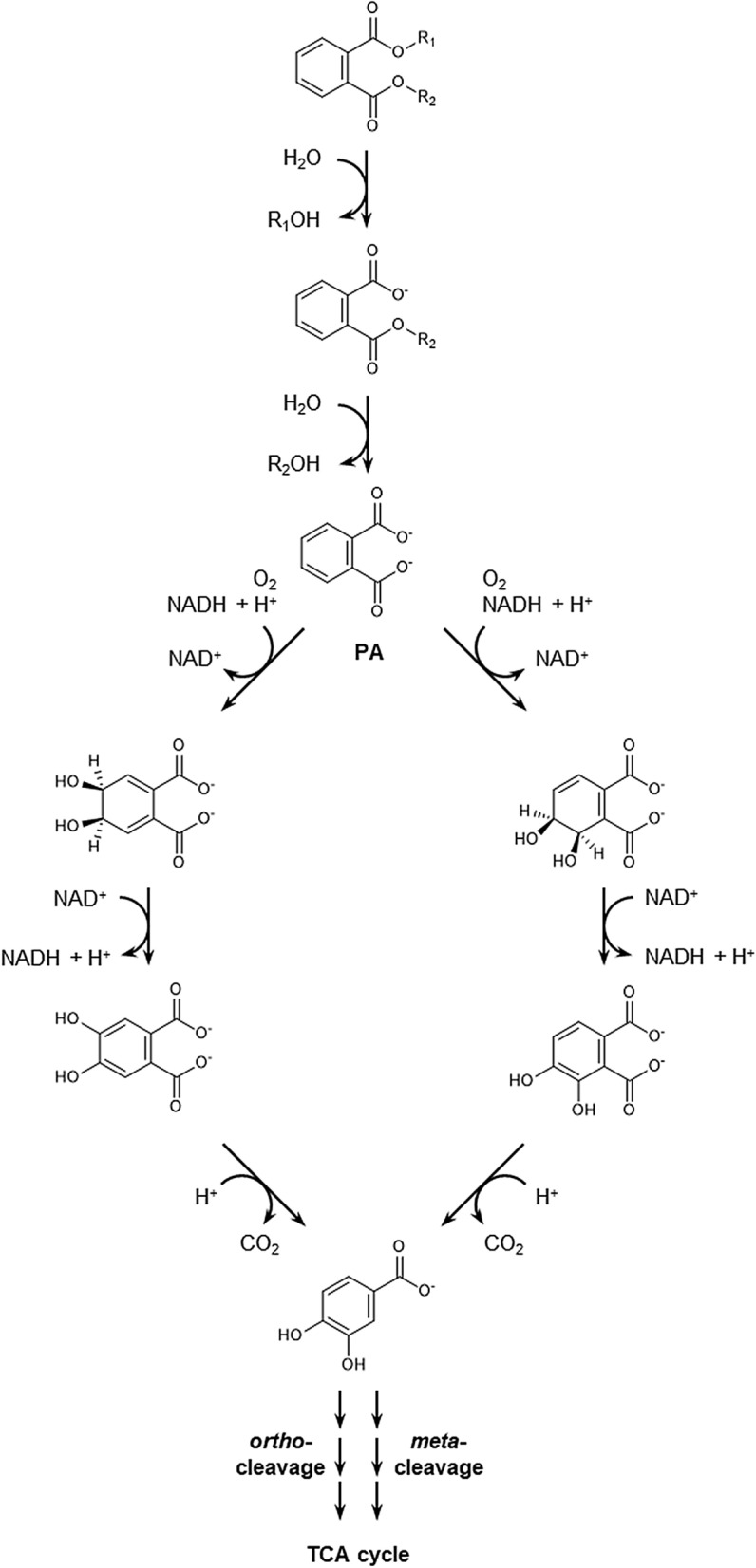
Degradation pathways of PAEs in aerobic bacteria. R_1_ and R_2_ = variable alkyl chains including methyl, ethyl, butyl, isobutyl, benzyl and 2-ethylhexyl functionalities. The first and second hydrolysis of the ester bonds may occur simultaneously without the formation of a monoalkyl phthalate intermediate

Among PAEs, di-*n*-butyl phthalate (DBP) is one of the most extensively applied plasticizers incorporated into high-molecular weight polymers such as polyvinyl chloride (PVC), and it is categorized as a high priority chemical according to a recent risk evaluation report of the US Environmental Protection Agency under the Toxic Substances Control Act (https://www.epa.gov/assessing-and-managing-chemicals-under-tsca). DBP has been found in landfill leachates, sediments, water, air, soils, plants, gas, and indoor dust (reviewed in Gao and Wen 2016; Chowdhary et al. 2018; Gao et al. 2018). Biodegradation is the major process for eliminating DBP from the environment, and the use of DBP as a carbon and energy source has been described for many aerobic bacterial strains from various ecosystems including *Pseudomonas fluorescens* B-1 (Xu et al. 2005), *Pseudomonas* sp. V21b (Kumar et al. 2017), *Pseudomonas* sp. YJB6 (Feng et al. 2021), *Paracoccus kondratievae* BJQ00001 (Xu et al. 2020), *Bacillus subtilis* sp. (Huang et al. 2018), *B. amyloliquefaciens* JR20 (Yuan et al. 2019), *Achromobacter* sp. (Jin et al. 2015; Benjamin et al. 2016), *Rhodococcus* sp. (Lu et al. 2009; Jin et al. 2010), *Arthrobacter* sp. ZH_2_ (Wang et al. 2012), *Gordonia* sp. (Wu et al. 2011a), *Delftia* sp. TBKNP-05 (Patil et al. 2006), *Agrobacterium* sp. (Wu et al. 2011b), *Sphingobium* sp. SM42 (Sungkeeree et al. 2016), *S. yanoikuyae* strain P4 (Mahajan et al. 2019), *Acinetobacter* sp. Strain LMB-5 (Fang et al. 2017), *Providencia* sp. 2D (Zhao et al. 2016), *Methylobacillus* sp. (Kumar and Maitra 2016), *Ochrobactrum* (Wu et al. 2010), and *Comamonas* sp. 51F (Kumar et al. 2017). Biodegradation of PAEs is usually initiated by the hydrolysis to phthalic acid (PA) and the respective alcohols catalyzed by intra- or extracellular esterases, often via monoalkylated intermediates (Niazi et al. 2001; Maruyama et al. 2005). In aerobic microorganisms, PA is most often converted to the central intermediate protocatechuate (3,4-dihydroxybenzoate) by two-component dioxygenases, either via 4,5-dihydro-*cis*-4,5-dihydroxy-PA (mostly in Gram-negative bacteria) or 3,4-dihydro-*cis*-3,4-dihydroxy-PA intermediates (mostly in Gram-positive bacteria) (Gao and Wen 2016; Boll et al. 2020) (Fig. 1).

The Hyrcanian Forests stretch 850 km along the southern coast of the Caspian Sea with a history dating back 25–50 million years. In 2019, they have been registered as a UNESCO World Heritage Site with a remarkable biodiversity (https://whc.unesco.org/en/list/1584/). The Saravan landfill is located inside Hyrcanian Forests 20 km outside of Rasht, Iran. With 800 tons of solid waste input per day, it severely endangers the ecosystem. Approximately 7 L s^–1^ of leachate are discharged from the Saravan landfill site to the soil, river, and groundwater. This leachate contains a considerable amount of heavy metals and numerous toxic organic compounds that seep into the international Anzali wetland near the Caspian Sea causing severe environmental problems. Plastics are second most abundant waste behind food waste in terms of incoming solid material to Saravan landfill (Shariatmadari et al. 2018; Karimpour-Fard 2019).

Here, we have evaluated bacterial degradation of PAE-derived plasticizers at the Saravan landfill. For this purpose, we established DBP-degrading enrichment cultures from which a *Paenarthrobacter* sp. was isolated with the capability of using many PAEs and *o*-phthalate as a sole source of energy and carbon. The study is accompanied by in vitro esterase assays and mass spectrometry-based analyses of degradation intermediates.

## Material and methods

### Chemicals

Monobutyl phthalate (MBP, 99% purity), di-*n*-butyl phthalate (DBP, 99%), diethylhexyl phthalate (DEHP, 99.5%), diethyl phthalate (DEP, 99%), dimethyl phthalate (DMP, 99%), phthalic acid (PA, > 99.5%), 2-ethyl-1-hexanol (99.6%), terephthalic acid (98%), and isophthalic acid (99%) were purchased from Sigma Aldrich Chemie GmbH (Hamburg, Germany). Acetonitrile, ethyl acetate, hexane, and formic acid were of HPLC grade and purchased from Carl Roth (Karlsruhe, Germany).

### Study area and sampling

Saravan landfill is located in the area of Rasht city, between the transit road of Rasht-Tehran and Siahrood River at an altitude of 50–250 m above sea level. More than 700 tons of waste are buried daily in this area in an unprincipled approach for 30 years. The vast and continuous flow of waste dumped at the site has led to an accumulation of waste up to 70 m (Karimpour-Fard 2019). Saravan waste leachate enters Anzali wetland and the Caspian Sea through Zarjub, Sefidrood, and Pirbazar rivers. Other environmental hazards include leachate penetration into the lower layers of the soil and groundwater contamination, atmospheric gas emissions, native and indigenous animals feeding on the wastes, and damages to vegetation and wildlife (Shariatmadari et al. 2018). The sampling was performed at a depth of 0–10 cm in three downstream areas of the landfill site (leachate passing site, longitude 378,296, latitude 4,105,555). In addition, samples were taken from an downstream area of the landfill site, which was not exposed to the leachate pollution (longitude 378,243, latitude 4,105,915).

### Measurement of some physical and chemical properties and PAEs of soil

Soil samples were air-dried, passed through a 2 mm sieve, and then dried in an oven at 105 °C for 10 h. Soil organic matter was measured by the Walkley–Black titration method. Electrical conductivity and the soil pH were measured in a 1:1 (soil: water) extract by an EC meter (JENWAY 4320), and pH meter (UNICAM 9455) respectively. For measurement of PAEs in soil samples, dioctyl adipate (DOA) as an internal standard was added to 1 g of the freeze-dried soil sample and 10 mL methanol was added. The mixture was vortexed (5 min), sonicated (30 min) and centrifuged (5 min at 3500 rpm). Subsequently, 5 mL of the upper phase was removed, followed by addition of 1 mL hexane after which the mixture was vortexed (2 min). Finally, 1 μL of the extracted hexane phase was injected into a gas chromatograph coupled to a triple quadrupole mass spectrometer detector (GC–MS, 7890A, Agilent) for PAEs determination. Chromatographic determination of PAEs was performed using a DB-5 ms column (J&W Scientific). The initial temperature of the column was 80 °C (2 min), then increased to 285 °C with 7 °C/min and was kept at 285 °C for 7 min. Helium gas was used as a carrier gas and splitless mode was used for sample injection. The GC injector and the MSD transfer line temperatures were set to 290 °C, and the ion source and quadrupole analyzer temperatures were adjusted to 230 and 150 °C, respectively**.**

### Enrichment of a microbial consortium degrading PAEs

The enrichment procedure was adapted from Jin et al. (2015) with slight modifications. A mineral salt medium (MSM) consisting of CaCl_2_ 0.01 g L^−1^, MgSO_4_ 0.1 g L^−1^, KH_2_PO_4_ 4.5 g L^−1^, (NH_4_)_2_ SO_4_ 1 g L^−1^, NaCl 1 g L^−1^, K_2_HPO_4_ 5.8 g L^−1^, and SL10 trace elements, pH 7, was used for enrichment and isolation. Contaminated soil (5 g) was mixed with 50 mL of sterile MSM in an Erlenmeyer flask with 100 mg L^–1^ of DEHP, DBP, DEP, DMP, respectively. The medium was incubated at 30 °C and 180 rpm for 7 days. 1 mL of the suspension was added to a fresh MSM containing 200 mg L^–1^ of PAEs and was incubated again for 1 week. Dependent on bacterial growth, this process was continued by increasing the amount of PAEs stepwise up to 1000 mg L^−1^ in the media. Four and eight weeks after incubation, the capability of the consortium to remove PAEs was evaluated via GC–MS analysis as described above and compared with controls without soil addition.

### Isolation and identification of bacterial DBP degraders

A DBP degrading bacterium was isolated on solid medium using MSM agar supplemented with 5 µL DBP (distributed on the agar surface); as a control, plates without a carbon source were prepared. The 16S rRNA gene was directly amplified from bacterial suspension using the bacterial primers 27F (5′-AGAGTTTGATCCTGGCTCAG-3′) and 1492R (5′-GGTTACCTTGTTACGACTT-3′). The 50 µL PCR reaction contained 25 µL Red Tag polymerase (Genaxxon bioscience, Ulm, Germany), 2.5 µL forward primer (10 µM stock solution), 2.5 µL reverse primers (10 µM stock solution), 20 µL sterile distilled water and a small amount of the pure colony. PCR mixture was applied to a temperature cycler (Flex cycler, Analytik Jena, Jena, Germany). The program used for PCR consisted of an initial denaturation step at 95 °C for 5 min, followed by 34 cycles at 95 °C for 30 s, 55 °C for 30 s, and 72 °C for 90 s, plus a final step at 72 °C for 5 min. The PCR product was evaluated by 1% agarose gel electrophoresis, quantified by a NanoDrop (PeqLab, Erlangen, Germany), and sequenced by Sanger sequencing (Eurofins Genomics Germany, Ebersberg, Germany).

### Transmission electron microscopy

Exponentially grown cells with 1000 mg L^–1^ DBP as carbon and energy source were adsorbed to glow-discharged Formvar-carbon-coated grids by placing 10 µL of the cell-suspension on the grid for 5 min. Samples were washed three times by touching the surface of the grid with drops of distilled H_2_O, and negatively stained for 30 s with 2% (wt/vol)uranyl acetate. Cells were imaged at 100 keV using a Hitachi HT7800 TEM coupled to a Xarosa Emsis camera.

### Substrate utilization assays

DBP, DEHP, DEP, DMP, MBP (200 mg L^–1^), 2-ethyl hexanol (100 mg L^–1^), isophthalic acid (5 mM), terephthalic acid (5 mM), and *ortho*-phthalic acid (10 mM) were used as sole carbon sources to test the capability of strain Shss to grow with these compounds in the same mineral salt medium as described above for the enrichment of the culture. The growth of strain Shss was measured at a wavelength of 578 nm by a UV-Ultrospec 3000 pro spectrophotometer (Pharmacia, Uppsala, Sweden).

### Biodegradation assay of DBP by strain Shss and identification of downstream products

For measuring the biodegradation of DBP by strain Shss, cells from an exponentially growing pre-culture were added to 10 mL medium containing 1000 mg (approximately 3.7 mM) DBP. The DBP had been added to the mineral salt medium described from a 100-fold stock solution in methanol. In control experiments, it was verified that methanol did not serve as carbon source. The cultures were incubated at 30 °C and 150 rpm. For analysis of metabolites, the entire culture was first acidified with 1 mL 1 M HCl followed by the extraction with an equal volume of ethyl acetate. The ethyl acetate phase was then diluted 1:4 (v/v) with methanol. Detection and identification of DBP and metabolites of its degradation were performed with an Acquity ultra performance liquid chromatography (UPLC®, Waters, MA) system coupled to a photodiode array detector. The chromatographic separation was performed using a BEH-C_18_ column (2.1 × 100 mm, 1.7 μm; Waters, MA, USA) at 30 °C and an acetonitrile (solvent A) and 10 mM ammonium formate pH 3 (solvent B) gradient at a flow rate of 0.3 mL min^–1^. The separation was accomplished by increasing the amount of solvent A from 5 to 100% within 4 min. DBP, MBP and PA were identified by their UV absorbance at 275 nm at a retention time of 5.3, 4.2, and 3.6 min, respectively. Chromatograms were analyzed using the Empower 3 (Waters, MA, USA) software. The residual concentrations of DBP and its intermediates in the liquid culture were calculated by extrapolating the peak area with standard curves.

### Identification of MBP by UPLC-ESI-QTOF-MS/MS

To confirm the nature of the intermediate identified via UPLC analysis, samples were analyzed after UPLC separation and electrospray ionization (ESI) by a quadruple time of flight MS device (ESI-Q-TOF–MS, Synapt G2-Si, Waters). Target compounds were separated using a BEH C_18_ column as described above. MS/MS analysis was performed at full scan mode in the m/z range of 50–1200 using an electrospray ionization (ESI) in negative mode with a capillary voltage of 1.5 kV, a source temperature of 120 °C, desolvation gas temperature of 500 °C, a gas flow of 800 L h^−1^ N_2_, and a cone gas flow of 50 L h^−1^ N_2_. Collision induced dissociation of precursor ions was performed by ramping the collision energy from 10 to 40 V. Ions with an m/z of 221.08 (± 0.01) and 165.02 (± 0.01) were monitored to identify MBP and PA, respectively. The data analysis was performed using Mass Lynx V4.1. The intermediates were identified by the mass pattern of each compound at the retention time of authentic standards.

### Growth with *o*-phthalic acid

To investigate the biodegradation of *o*-phthalic acid (PA) by strain Shss, 0.5 mL of an exponentially growing Shss pre-culture (OD_578_ = 0.5) was inoculated in MSM supplemented with 12 mM PA. The culture was incubated at 30 °C and 180 rpm for 2 days. The growth of strain Shss was monitored at OD_578_. After acidification of the culture with 1 M HCl to pH 2, the PA concentration was determined photometrically at 276 nm (ε = 1200 mol^−1^ cm^−1^) (Ebenau-Jehle et al. 2017).

### In vitro DBP and MBP esterase assays

Cells were grown in 2-L-Erlenmeyer flasks containing 800 mL mineral salt medium media described above with 3.7 mM DBP (30 °C) while shaking at 150 rpm. For measuring intracellular esterase activities, cells at OD_578nm_ 0.5 were centrifuged (20 min, 6000 rpm, PTI F7S), washed four times with media without substrate (20 min, 6000 rpm, Hermle A8.24) and suspended in 3 mL of the remaining media. DNase was added before passing the suspension twice through a French pressure cell at 5.4 MPa. The crude extract was centrifuged at 100,000×*g* for 1 h. The resulting supernatant was used for the enzyme assays; supernatant heated for 15 min at 95 °C served as a negative control. For testing possible extracellular esterase activities, culture supernatant was 100-fold concentrated by ultrafiltration (10 kDa, Amicon cell, model 8200, Amicon) prior to use in assays.

Assays for analyzing intracellular esterase activities contained 930 µL medium, 10 µL 2 M Tris/HCl pH 8.8 (resulting in final pH 7.2), and 50 µL cell extract. In assays for extracellular esterase activities, 990 µL the 100-fold concentrated culture medium was used directly. Both assays were started by the addition of 10 µL of a 100 mM DBP stock solution in methanol. Samples of 100 µL were taken at different time points by adding 400 µL 0.1% formic acid in methanol. After centrifugation (20 min, 14,000 rpm), samples were analyzed by UPLC as described above, but the solvent system was modified to 0.1 formic acid in methanol (solvent A) and 0.1% formic acid in water (solvent B). DBP, MBP and PA were identified at 275 nm at a retention time 5.6, 4.9 and 4.0 min respectively. Specific activities were defined as nmol DBP or MBP consumed per minute per mg protein in the extract/supernatant Protein concentration was determined by the Bradford method.

## Results and discussion

### Physical and chemical properties and PAEs concentration of soil from Saravan landfill

Table 1 shows the concentrations of various PAEs and data from physicochemical analyses in soil samples taken downstream and upstream of the leachate of the Saravan landfill. The 3.4-fold higher electric conductivity of the downstream samples is assigned to leachate components. In accordance with previous studies from other sites (Net et al. 2015; Gao and Wen 2016; Gao et al. 2018), DEHP and DBP were the major PAE components found in the soil samples from Saravan landfill. They were 4.5- and 10-fold above the ecological risk limit (ERL) values of DEHP (1 mg kg^–1^) and DBP (0.7 mg kg^–1^), respectively (Net et al. 2015). The fivefold higher concentration of DEHP vs DBP is assigned to its generally lower biodegradation rates resulting in a higher persistence at the landfill (Net et al. 2015).

**Table 1:** Physicochemical properties and PAE concentrations of samples taken from Saravan landfill

Property	Exposed to leachate	Not exposed to leachate
pH	8.2	7.9
Electric conductivity (dS m^−1^)	0.825	0.242
Clay/silt/sand (%)	28/28/44	41/38/21
Organic matter (%)	1.64	2.31
Dimethylphthalate (mg kg^−1^)	< 0.01	< 0.01
Diethylphthalate (mg kg^−1^)	< 0.01	< 0.01
Benzyl butyl phthalate (mg kg^−1^)	0.447	0.462
Di-*n*-butylphthalate (mg kg^−1^)	0.765	0.672
Diisobutylphthalate (mg kg^−1^)	0.082	0.065
Diethylhexyl phthalate (mg kg^−1^)	4.51	0.404
Total PAE (mg kg^−1^)	5.804	1.603

### Establishment of a PAE-degrading bacterial enrichment culture

**Fig. 2:**
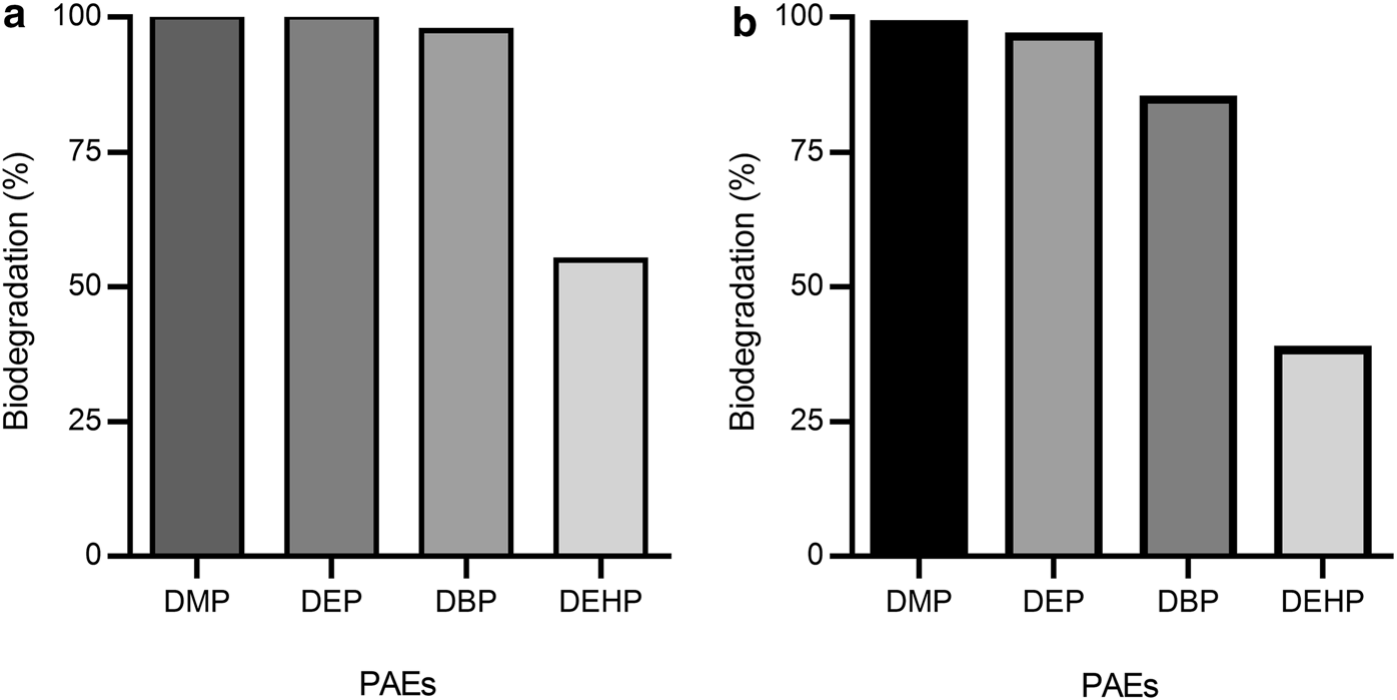
Biodegradation of PAEs by the enrichment culture SA1 established from Saravan landfill leachate. **a** After 4 (400 mg L^–1^ substrate) and **b** 8 weeks (1000 mg L^–1^ substrate) of cultivation. 100% refers to the complete removal of PAEs

For establishing a PAE degrading bacterial enrichment culture, the soil exposed to leachate was incubated with a mixture of PAEs including DMP, DEP, DBP, and DEHP. For the adaptation of the culture, the concentration of the individual PAEs was stepwise increased from 100 to 1000 mg L^–1^. Growth was determined by OD_578_ measurements and the consumption of individual PAEs was monitored by GC–MS analyses. When incubated with 400 mg L^–1^ PAEs, DMP, DEP, and DBP were degraded almost completely within 4 weeks, whereas 45% of the added DEHP remained (Fig. 2a). The consortium degraded 98.8%, 96.4%, 84.7%, and 38.3% of 1000 mg L^–1^ DMP, DEP, DBP, and DEHP, respectively (Fig. 2b). Degradation was accompanied by an increase of OD_578_ between 0.8 and 1.25. In a control without PAE addition, OD_578_ remained at 0.07 and 0.075, respectively, indicating that the increase of OD_578_ correlates with PAE consumption. The observation of the lower degradation rate of DEHP vs PAEs with smaller alkyl chains has frequently been observed. The preferred degradation of DMP, DEP, and DBP vs DEHP reflects their low concentrations in the landfill leachate. In contrast, the residual high concentration of DEHP (4.51 mg kg^–1^) is assigned to the lower biodegradation rate of this PAE.

### Isolation of PAE-degrading bacterial strains

**Fig. 3:**
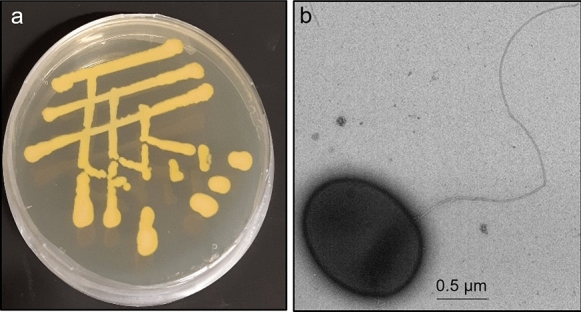
*Paenarthrobacter* sp. strain Shss isolated in this work. **a** Growth on LB-agar. **b** Transmission electron microscopy image of a exponentially grown cell in liquid medium with 1 g L^–1^ DBP, contrasted by negative staining

From the PAE-degrading enrichment culture established from Saravan landfill leachate, attempts to isolate bacterial strains using PAEs as carbon and energy source under aerobic conditions were performed using an agar-based solid medium. After 2 months of incubation, the formation of orange colonies was observed in a mineral salt medium containing agar and DBP (5 µL distributed on the agar surface) as only carbon and energy source; similar colonies were formed in LB medium (Fig. 3a). The cells of the strain referred to as strain Shss were rod-shaped/coccoidal with an average diameter × length ≈ 0.7 µm × 1.2 µm (Fig. 3b). The strain was Gram-positive, not spore-producing, catalase-positive, motile, and contained a single flagellum (Fig. 3b). The 16S rRNA gene was amplified by colony PCR. BLAST analysis of the sequence obtained (1068 bp) revealed 99.44% identity to *Paenarthrobacter ureafaciens* strain NC (DSM 20126). The partial 16S rRNA gene sequence of *Paenarthrobacter* sp. strain Shss was registered in NCBI GenBank with the accession number MN310736.

*Paenarthrobacter* strain Shss utilized DBP, MBP, DEP, DMP (200 mg L^–1^), and PA (5 mM) as the sole source of carbon and, together with O_2_, as an energy source (OD_578_ reached were ≥ 0.3 during growth with each substrate). It did not utilize DEHP, MEHP (200 mg L^–1^, each), 2-ethyl hexanol (200 mg L^–1^), terephthalic and isophthalic acid (5 mM each).

### Analysis of culture intermediates during DBP degradation by *Paenarthrobacter* strain Shss

**Fig. 4:**
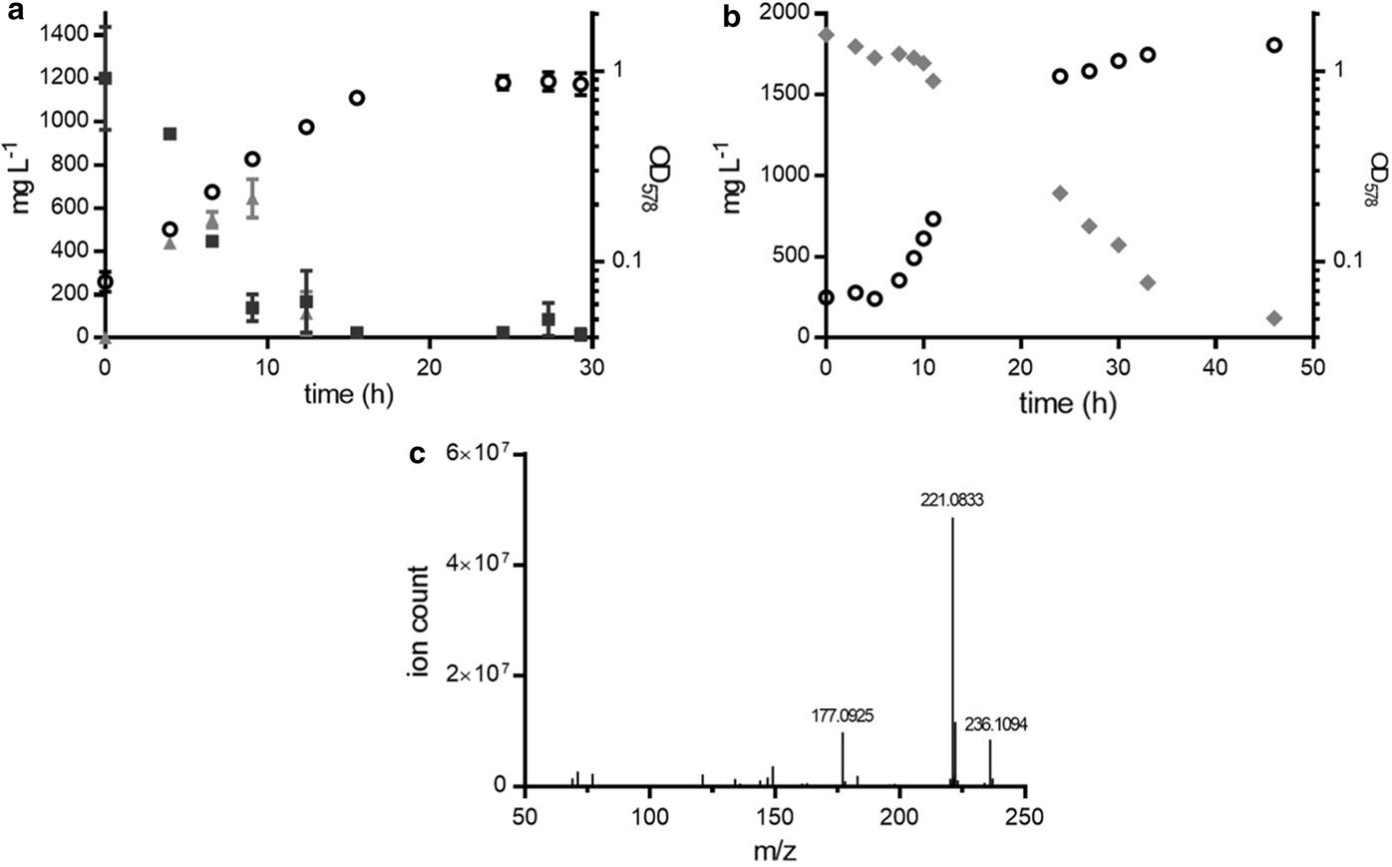
Growth of *Paenarthrobacter* strain Shss with DBP and PA. **a** Growth curve with DBP as determined by   OD_578_, the consumption of ■ DBP, and the transient formation of ▲MBP. **b** Growth curve with PA as determined by OD_578_ measurements and the consumption of PA. **c** Ion chromatogram of MBP as determined by UPLC-ESI-QTOF-MS

The growth of *Paenarthrobacter* strain Shss with DBP and the formation of intermediates in the culture medium were investigated. For this purpose, the culture supernatants were analyzed by UPLC coupled to photodiode array detection at different OD_587_ values reached. DBP (1000 mg L^–1^) was almost completely degraded within 15 h coupled to an increase of OD_578_ to around 1.0 (Fig. 4a). While DBP continuously decreased in the course of the growth experiment, a product increased within the first 10 h of incubation. In the following 5 h, it was completely consumed indicating that it represents a transiently accumulating intermediate during DBP degradation. The intermediate was subjected to UPLC analysis coupled to ESI-QTOF-MS detection. It co-eluted with an authentic MBP standard, and the monitored ions m/z 221.08 ± 0.01 and 165.02 ± 0.01 (negative mode) were fitted to those of the standard and the calculated mass (Fig. 4c).

The results obtained suggest that DBP was degraded via MBP to one PA and two molecules *n*-butanol. As PA was not detected at significant concentrations in the medium, it was suggested that it was readily further degraded by intracellular enzymes. To confirm this assumption, growth of *Paenarthrobacter* strain Shss with 2000 mg L^–1^ PA as only carbon and energy source was investigated. Indeed, it readily converted PA under aerobic conditions with approximately 1000 mg L^–1^ of PA being consumed within 15 h (Fig. 4b). Thus, DBP and PA are apparently degraded at similar rates suggesting that hydrolysis of DBP to PA via MPB is not rate limiting during complete DBP degradation.

The complete degradation of 1000 mg L^–1^ DBP within 15 h by strain *Paenarthrobacter* strain Shss was combined with a growth to OD_578_ ≈ 1.0. The very low doubling time perfectly correlated with the half-life of DBC (both 5 h). These values place *Paenarthrobacter* strain Shss among the most efficient DBP degrading microorganisms known to date. In Table 2, a compilation of reports on microbial DBP degradation at high concentrations (threshold set to ≥ 400 mg L^–1^) is presented; in addition, studies that reported on the degradation on DBP concentrations above 1000 mg L^–1^ are further discussed below. Notably, a direct comparison of degradation rates determined in individual studies has to be taken cautiously, as the studies have often been conducted under different conditions.

**Table 2:** Bacterial strains reported to degrade DBP at concentrations ≥ 400 mg mL^–1^

Strain	DBP degradation capability	References
*Achromobacter denitrificans* SP I	2780 mg L^–1^ in 24 h	(Benjamin et al. 2016)
Paenarthrobacter strain Shss	1000 mg L^–1^ in 15 h	This work
*Sphingobium yanoikuyae* strain P4	1000 mg L^–1^ in 24 h	(Mahajan et al. 2019)
*Rhodococcus* sp. JDC-11	900 mg L^–1^ in 24 h	(Jin et al. 2010)
*Delftia* sp. TBKNP-05	2780 mg L^–1^ in 120 h	(Patil et al. 2006)
*Pseudomonas* sp. YJB6	1600 mg L^–1^ in 120 h	(Feng et al. 2021)
*Gordonia* sp. QH-11	750 mg L^–1^ in 45 ha	(Jin et al. 2012)
*Gordonia* sp.	400 mg L^–1^ in 30 h	(Wu et al. 2011a)
*Arthrobacter* sp. strain ZH2	500 mg L^–1^ in 48 h	(Wang et al. 2012)
*Methylobacillus* sp. V29b	1400 mg L^–1^ in 192 h^b^	(Kumar and Maitra 2016)
*Ochrobactrum* sp. JDC-41	500 mg L^–1^ in 72 h	(Wu et al. 2010)
*Acinetobacter* sp. strain LMB-5	400 mg L^–1^ in 60 h	(Fang et al. 2017)
*Pseudomonas* sp. V21b *Comamonas* sp. 51F	1200 mg L^–1^ in 192 h^b^ 900 mg L^–1^ in 192 h^b^	(Kumar et al. 2017)

Strains that degrade DBP at concentrations ≥ 1000 mg mL^–1^ are further discussed in the text

^a^With lag phase

^b^Of 2000 mg L^−1^ initial concentration

In *Achromrobacter denitrificans* SPI, the complete consumption of 2780 mg L^–1^ (10 mM) within 24 h appears to be the highest rate reported so far, however, no quantitative correlation between biomass production and DBP consumption was presented (Benjamin et al. 2016). The degradation of 1000 mg DBP L^–1^ in 12 h in *S. yanoikuyae* strain P4 was accompanied by growth to OD_600_ = 1.4 (Mahajan et al. 2019), which is in the range of the values obtained with *Paenarthrobacter* strain Shss. In *Rhodococcus* sp. JDC-11, the complete degradation of 1000 mg L^–1^ was achieved within 24 h, however the biomass formed during this process was not reported (Jin et al. 2010). *Delftia* sp. TBKNP-05 completely degraded 2780 mg L^–1^ in 120 h and reached an OD_660_ of 1.4 (Patil et al. 2006), whereas *Methylobacillus* sp. V29b degraded 1400 mg in 192 h, thereby only growing to OD_600_ = 0.3 (Kumar and Maitra 2016). In *Pseudomonas* sp. YJB6, the degradation of 1600 mg L^–1^ DBP was accompanied with an increase of OD_600_ around 1.0 (Feng et al., 2021). In contrast, only very low OD_600_ values of around 0.16 were reported in *Pseudomonas* strain V21b and *Comamonas* sp. 51F during the 192 h degradation of 1200 mg L^–1^ DBP and 900 mg L^–1^, respectively (Kumar et al. 2017). These examples reflect that the correlation of biomass formation and DBP degradation varies strongly in these studies, indicating either largely differing DBP assimilation capacities of individual strains and/or technical differences in analyzing growth and DBP biodegradation. In summary, among the strains reported to degrade DBP at concentrations higher than 1000 mg L^–1^, *Paenarthrobacter* strain Shss appears to be highly efficient in terms of degradation rate and assimilation into biomass.

### Cellular esterases are involved in DBP degradation in *Paenarthrobacter* sp. strain Shss

**Fig. 5:**
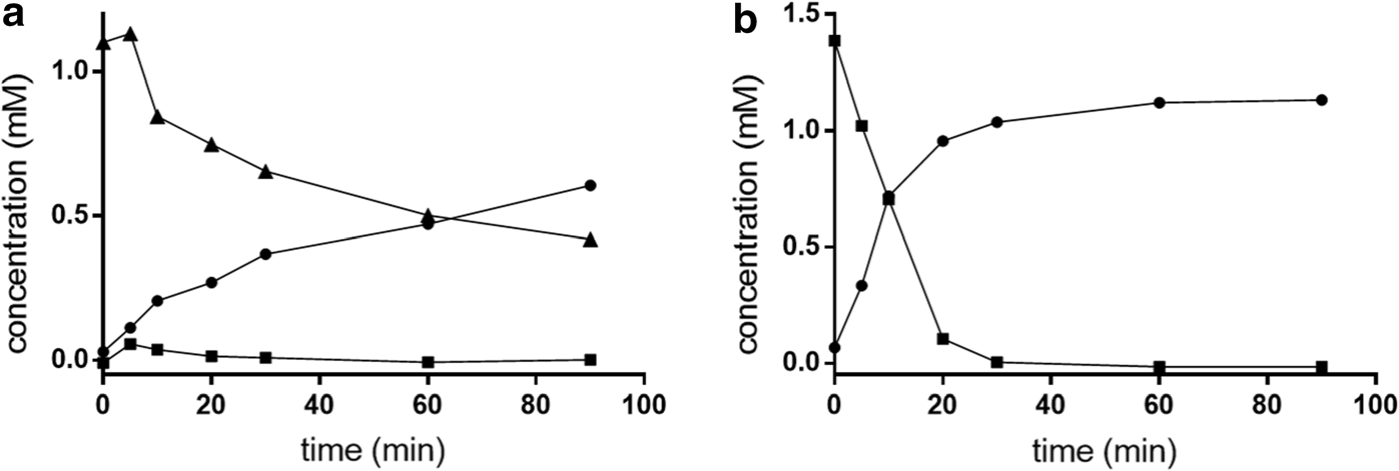
In vitro esterase assay with DBP and MBP as substrates. **a** Consumption of DBP (▲), and the formation of MBP (■) and PA (●) in cell extracts of *Paenarthrobacter* strain Shss. **b** Degradation of MBP (■) and formation of PA (●) in cell extracts. Background PA concentrations present in cell extracts without addition of DBP (**a**) or MBP (**b**) were subtracted from the values determined

UPLC-based analyses of culture supernatants during the degradation of DBP by *Paenarthrobacter* strain Shss suggested MBP as an intermediate during DBP degradation. To determine whether this accumulation was due to extracellular or cellular esterases, cells were grown on the 800-mL-scale to OD_578nm_ ≈ 0.5. Samples from these cultures were separated in cell-free culture supernatants and entire cells by centrifugation. Both fractions were analyzed by UPLC for MBP and MBP consumption and PA formation. Using 100-fold concentrated culture supernatants in the assays, virtually neither DBP nor MBP esterase activities were monitored. In contrast, using cell extracts from washed cells, the time-dependent consumption of DBP and the formation of MBP was monitored at a specific rate of 104 nmol DBP consumed min^–1^ mg (protein)^–1^ (Fig. 5a). In heat-treated controls, no DBP esterase activity was observed. When MBP replaced DBP as substrate in assays with cell extracts, the ready conversion to PA was observed at a specific rate of 295 nmol MBP consumed min^–1^ mg (protein)^–1^ (Fig. 5b). These results indicate that both, DBP and MBP hydrolysis are catalyzed by cellular esterases. The transient accumulation of MBP in the culture medium suggests that DBP esterase may be attached to the outer leaflet of the cytoplasmic membrane.

The sequential hydrolysis of PAEs via monoalkylated intermediates as shown for *Paenarthrobacter* strain Shss has been described for other PAE-degrading organisms (Akita et al. 2001; Maruyama et al. 2005; Lu et al. 2009), and cellular esterases specific for monoalkylated PAEs have been reported (Nishioka et al. 2006; Hara et al. 2010; Huang et al. 2019). Though PAE esterases have been reported to have a cellular location, little is known about their subcellular location, and it is unknown whether PAE esterases are associated with the cytoplasmic membrane. Clear evidence for extracellular esterases involved in PAE degradation is lacking.

## Conclusion

In this work the previously unknown capacity of the genus *Paenarthrobacter* for the degradation of PAEs was identified in an isolate from a highly PAE-contaminated Iranian landfill. It proceeds via the stepwise hydrolysis of the two ester bonds by two highly active cellular esterases followed by the consumption of the PA formed. The esterases are highly efficient as the growth rate with DBP and PA was similar suggesting that complete degradation of PA rather than the conversion DBP to PA appears to be rate-limiting.

The degradation capacity of *Paenarthrobacter* strain Ssh is amongst the fastest reported so far. Consequently, we propose that *Paenarthrobacter* strain Shss plays a previously overseen important role in the elimination of DBP from contaminated soil. Due to its rapid growth and high degradation capacities it might serve as a promising candidate strain for PAE degradion in engineered systems.
